# Methamphetamine-Associated Cardiomyopathy and Cardioembolic Stroke: Brain–Heart–Gut Axis Crosstalk, Diagnostic Strategies, and Anticoagulation Challenges

**DOI:** 10.3390/ijms262411908

**Published:** 2025-12-10

**Authors:** Pei-Jung Lin, Chia-Hui Wu, Jen-Hung Huang, Jakir Hossain Bhuiyan Masud, Chien-Tai Hong, Lung Chan, Chen-Chih Chung

**Affiliations:** 1 Department of Neurology, Taipei Medical University—Shuang Ho Hospital, New Taipei 235, Taiwan; 23348@s.tmu.edu.tw (P.-J.L.); 11331@s.tmu.edu.tw (C.-H.W.); ct.hong@tmu.edu.tw (C.-T.H.); cjustinmd@gmail.com (L.C.); 2 Taipei Neuroscience Institute, Taipei Medical University—Shuang Ho Hospital, New Taipei 235, Taiwan; 3 Division of Cardiovascular Medicine, Department of Internal Medicine, Wan Fang Hospital, Taipei Medical University, Taipei 116, Taiwan; b8601022@tmu.edu.tw; 4 Division of Cardiology, Department of Internal Medicine, School of Medicine, College of Medicine, Taipei Medical University, Taipei 110, Taiwan; 5 Taipei Heart Institute, Taipei Medical University, Taipei 110, Taiwan; 6 Department of Biomedical Informatics and Data Science, The University of Alabama at Birmingham, Birmingham, AL 35294, USA; jmasud@uabmc.edu; 7 Department of Neurology, School of Medicine, College of Medicine, Taipei Medical University, Taipei 110, Taiwan

**Keywords:** anticoagulation, brain–heart–gut axis, cardiomyopathy, diagnosis, left ventricular thrombus, methamphetamine, stroke

## Abstract

Methamphetamine (MA) abuse has emerged as a multisystem insult driving cardiovascular and neurovascular consequences. Methamphetamine-associated cardiomyopathy (MACM) remains an underrecognized cause of cardioembolic stroke through left ventricular thrombus (LVT) formation. MA-induced gut dysbiosis and enteric neural disruption exacerbate systemic inflammation and autonomic imbalance, resulting in broader dysregulation of the brain–heart–gut axis. This study aimed to synthesize contemporary evidence on chronic MA exposure and its role in LVT formation, stroke pathogenesis, diagnostic approaches, and anticoagulation management. We conducted a focused narrative review of PubMed- and Scopus-indexed literature (1990–2025) addressing cardiovascular, neurovascular, and gut-mediated consequences of chronic MA exposure. Observational cohorts and case reports were integrated to characterize pathophysiology, imaging approaches, and therapeutic considerations, supplemented by a representative clinical case. Chronic MA exposure mediates persistent catecholamine excess, myocardial fibrosis, ventricular dysfunction, and a prothrombotic milieu. Gut dysbiosis-related inflammation and autonomic dysregulation further promote intracardiac stasis. Affected individuals are typically young men with severe systolic dysfunction (left ventricular ejection fraction 20–30%), with a substantial proportion demonstrating apical or mural LVT on systematic imaging. Case-level evidence highlights a broader systemic embolic burden, involving the limbs, kidneys, and aorta. Echocardiography remains the first-line screening method, while cardiac CT and MRI offer greater sensitivity for thrombus detection. Anticoagulation is challenged by bleeding risk, inconsistent adherence, and the absence of standardized protocols. MACM represents a critical and underrecognized etiology of cardioembolic stroke in young adults. Early recognition of brain–heart–gut axis disruption, systematic cardiac imaging, and individualized anticoagulation are crucial for preventing emboli. Prospective registries and standardized imaging-guided treatment strategies are needed to improve outcomes in this high-risk population.

## 1. Introduction

Methamphetamine (MA) remains one of the most widely abused psychostimulants worldwide, with increasing prevalence across high- and middle-income countries [1,2]. In Taiwan, MA is consistently the most frequently detected illicit substance, representing a persistent public health challenge [3,4]. Its systemic toxicity affects multiple organ systems, particularly the cardiovascular, cerebrovascular, and central nervous systems (CNSs) [5,6,7]. In addition to direct end-organ injury, MA induces complex neurocardiac interactions through dysregulation of the brain–heart–gut axis. Alterations in gut microbiome composition, impaired enteric nervous system (ENS) function, and systemic inflammation have been implicated in autonomic imbalance and pathological brain–heart–gut signaling, amplifying hemodynamic instability, arrhythmogenesis, and myocardial injury.

The incidence of ischemic stroke in younger adults (18–49 years) has risen substantially and accounts for approximately 15% of all cases [8]. Illicit drug use, particularly MA, is considered a key contributor due to its potent sympathomimetic effects, arrhythmogenic potential, and chronic cardiotoxicity [8,9,10]. While most existing research focuses on MA-induced neurovascular complications such as intracranial hemorrhage and vasospasm-related ischemia, a critical mechanism remains underrecognized: cardiogenic stroke resulting from left ventricular thrombus (LVT) secondary to methamphetamine-associated cardiomyopathy (MACM) [9].

MACM is a distinct subtype of toxic cardiomyopathy arising from chronic MA exposure. It is characterized by severe left ventricular systolic dysfunction, biventricular dilatation, and malignant arrhythmias, which collectively create a prothrombotic environment conducive to LVT formation [7]. Although MACM-related stroke may resemble atrial fibrillation (AF)–-associated stroke clinically, the underlying mechanisms differ fundamentally, reflecting a pathophysiologic continuum of autonomic dysregulation, sympathetic overactivation, and progressive myocardial remodeling. Despite increasing recognition of MACM and its association with ventricular thrombus and cardioembolic stroke, the mechanistic links between neuroautonomic dysregulation, myocardial remodeling, and thrombogenesis remain poorly synthesized.

**Figure 1:**
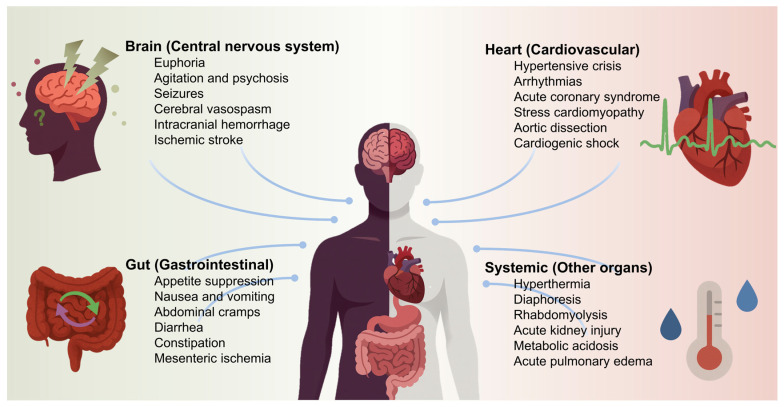
Acute MA toxicity across major organ systems. The schematic depicts the principal acute toxic effects of methamphetamine involving the central nervous, cardiovascular, gastrointestinal, and systemic organ systems.

This focused review synthesizes current evidence on brain–heart–gut axis disruption in MA users and its contribution to LVT formation and cardioembolic stroke. We outline relevant pathophysiological mechanisms, diagnostic challenges, and therapeutic considerations, and present a representative clinical case in a young adult. To contextualize the multi-organ impact of acute MA toxicity, Figure 1 summarizes its effects across the central nervous, cardiovascular, gastrointestinal, and systemic axes. These acute pathophysiologic perturbations initiate a cascade culminating in myocardial dysfunction, arrhythmogenesis, and prothrombotic states that predispose to stroke. By integrating mechanistic insights with clinical observations, this work aims to improve recognition, diagnostic precision, and management of this increasingly prevalent yet frequently overlooked manifestation of MA toxicity.

## 2. Pathophysiology of MACM and Thrombus Formation

MA induces structural and functional myocardial injury through a spectrum of mechanisms tightly coupled to dysregulation of the brain–heart axis. After systemic absorption, MA exerts potent sympathomimetic effects in the CNS, producing sustained tachycardia, hypertension, and increased myocardial oxygen demand [9]. Chronic exposure results in cardiomyocyte injury, oxidative stress, mitochondrial dysfunction, and progressive fibrosis, ultimately leading to ventricular dilatation and severe contractile impairment [7]. Deterioration of left ventricular systolic function and chamber enlargement promote intracavitary stasis. The left ventricular apex is particularly susceptible to thrombus formation because it physiologically experiences lower shear stress and reduced intracavitary blood-flow velocity [11]. When contractile dysfunction, such as akinesia or aneurysmal remodeling, is present, stasis is further accentuated [7,12]. Furthermore, in both stress-induced cardiomyopathy and MACM, catecholamine excess preferentially affects the apical segments, producing transient apical akinesia that amplifies local stasis and predisposes to LVT [13].

Combined with MA-induced endothelial activation, upregulation of procoagulant mediators such as tissue factor and plasminogen activator inhibitor-1 (PAI-1), and enhanced platelet aggregation, contributing to a prothrombotic state that increases the risk of LVT and cardioembolic stroke [7,9,14,15].

Comparative evidence suggests that MACM progresses more rapidly and diffusely than idiopathic or alcoholic cardiomyopathies, often with greater biventricular involvement and more pronounced reductions in ejection fraction [16]. Malignant arrhythmias and hemodynamic instability frequently occur, further increasing the risk of thromboembolism [5,16].

### 2.1. Role of CNS

MA is highly lipophilic and rapidly penetrates the blood–brain barrier, where it profoundly alters central monoaminergic neurotransmission. Acute exposure increases dopaminergic and noradrenergic signaling, producing heightened arousal, euphoria, and psychomotor activation [17,18]. In contrast, repeated or high-dose use depletes presynaptic monoamines and impairs striatal and limbic circuits [17].

In addition to neurotransmitter dysregulation, chronic MA exposure disrupts higher-order autonomic networks in the medial prefrontal cortex, anterior cingulate cortex, insular cortex, amygdala, hypothalamus, and brainstem autonomic nuclei [1,9,18]. Persistent activation of these regions produces chronic sympathetic hyperactivity, impaired cardiovascular rhythm regulation, and long-term hemodynamic instability [2,5,9]. This top-down dysregulation drives cycles of hyperexcitability and myocardial exhaustion, predisposing to arrhythmias, remodeling, and blood stasis.

### 2.2. Peripheral Nerve–Myocardium Interface

Excessive norepinephrine release from cardiac sympathetic terminals reinforces sympathetic predominance and diminishes parasympathetic tone, leading to autonomic imbalance and impaired electrophysiological stability [2,18,19]. Sustained β-adrenergic stimulation induces oxidative injury, mitochondrial dysfunction, and apoptosis in cardiomyocytes, accompanied by interstitial fibrosis and contractile decline [5,6]. Persistent peripheral sympathetic activation may additionally promote local inflammatory responses, further exacerbating myocardial vulnerability [5].

Damage at this peripheral interface represents an essential downstream component of brain–heart axis dysfunction. Initiated centrally and amplified peripherally, this process fosters electrical instability, left ventricular deterioration, and a prothrombotic substrate, providing a mechanistic link between MA exposure and cardiogenic stroke in MACM.

### 2.3. Bidirectional Disruption of the Brain–Heart Axis: From Central Autonomic Circuits to Cardiac Afferents

In MACM, myocardial dilatation, ischemia, and inflammation may abnormally activate vagal and spinal sensory fibers, altering neuroplasticity and autonomic regulation within brainstem centers [20]. Dysregulated afferent input alters baroreflex sensitivity (BRS) and autonomic regulation, leading to desensitization and increased arrhythmic susceptibility [2,19]. This bottom–up feedback further destabilizes central control, reinforcing sympathetic overactivity and creating a bidirectional failure of the brain–heart axis.

### 2.4. MA Effects on Gut Microbiota and ENS

Emerging evidence suggests that MA alters the composition of the gut microbiota and the ENS. Individuals with long-term MA use demonstrate reduced microbial diversity, characterized by the depletion of beneficial taxa, such as *Bacteroidaceae* and *Faecalibacterium*, and an increased abundance of pro-inflammatory taxa, including *Sphingomonadales*, *Romboutsia*, and *Lachnospiraceae* [21,22]. These compositional shifts are associated with impaired intestinal barrier integrity, which facilitates the translocation of endotoxins into the systemic circulation and amplifies inflammatory responses.

Beyond localized gut effects, MA influences CNS function through ENS and vagal afferent pathways. Animal and human studies have shown that MA downregulates tight junction proteins, increases circulating levels of inflammatory mediators such as lipopolysaccharide (LPS) and tumor necrosis factor-alpha (TNF-α), and potentially activates the gut–brain inflammatory axis [22,23,24].

Microbial metabolites derived from dysbiotic gut communities may contribute to heightened thrombotic risk. Among these, trimethylamine N-oxide (TMAO) has emerged as a key mediator. TMAO promotes vascular inflammation by upregulating pro-inflammatory cytokines and adhesion molecules, as well as activating the nucleotide-binding oligomerization domain-like receptor family pyrin domain-containing 3 (NLRP3) inflammasome, ultimately leading to endothelial dysfunction [25,26]. It also impairs antioxidant defense mechanisms and increases the production of reactive oxygen species (ROS), further exacerbating vascular injury [26,27]. TMAO concurrently enhances stimulus-dependent platelet activation by promoting intracellular Ca^2+^ release, thereby increasing platelet aggregation and accelerating thrombus formation [25,26,27]. These interconnected mechanisms place TMAO at the intersection of gut dysbiosis and cardiovascular complications in MACM.

### 2.5. Molecular Mechanisms of MA-Induced Myocardial Injury and Thrombogenesis

MA-induced myocardial injury arises from a multifaceted cascade involving mitochondrial dysfunction, oxidative stress, apoptotic signaling, and pro-fibrotic remodeling. Preclinical studies demonstrate that MA suppresses Sigma-1 receptor activity, reducing cAMP response element-binding protein-mediated transcription and downregulating mitochondrial fission protein-1. This impairs mitochondrial dynamics, disrupts the membrane potential, and diminishes ATP production, thereby increasing susceptibility to apoptosis [17,28].

In parallel, MA activates NADPH oxidase-2, markedly elevating ROS levels. Excess ROS further damages mitochondrial membranes and activates intrinsic apoptotic pathways, including the Bcl-2/Bax imbalance, cytochrome c release, and caspase-3 activation [1,17].

Chronic exposure also triggers pro-fibrotic molecular remodeling. MA upregulates periostin, α-smooth muscle actin, and collagen-synthesizing fibroblast markers, consistent with activation of trace amine-associated receptor-1/cAMP signaling pathways [1,7,17]. This results in the persistent deposition of extracellular matrix and the stiffening of the myocardial interstitium.

In addition to direct cardiomyocyte toxicity, MA exerts prothrombotic effects at the molecular level. Experimental models show that amphetamine derivatives increase endothelial tissue factor expression, suppress tissue factor pathway inhibitor activity, and elevate PAI-1 levels, shifting the coagulation cascade toward thrombogenesis [15].

MA-induced oxidative stress upregulates intercellular adhesion molecule-1 (ICAM-1) and vascular cell adhesion molecule-1 (VCAM-1), facilitating leukocyte adhesion and platelet–endothelial interactions that initiate thrombus formation [1].

Collectively, these alterations culminate in a maladaptive myocardial environment with impaired energetics, structural disarray, and heightened thrombogenic potential, establishing the pathophysiological foundation for LVT formation in MACM [1,17]

### 2.6. Integrated Pathophysiological Framework

**Figure 2:**
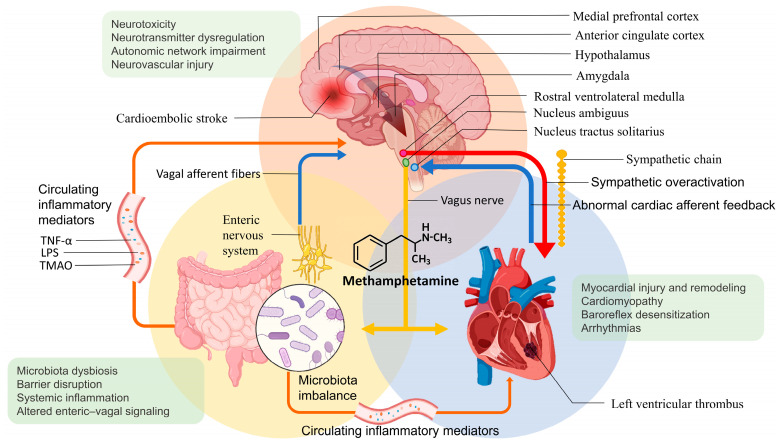
Proposed integrative model of brain–heart–gut axis dysregulation in chronic MA use to cardioembolic stroke. The schematic illustration depicts the multi-level neurocardiac and systemic pathways through which chronic MA exposure contributes to LVT formation and cardioembolic stroke. MA induces excessive release of dopamine and norepinephrine, leading to sustained activation of cortical (medial prefrontal and anterior cingulate cortices), subcortical (amygdala and hypothalamus), and brainstem autonomic centers (nucleus tractus solitarius, nucleus ambiguus, and rostral ventrolateral medulla). This central autonomic dysfunction drives sympathetic overexcitation and hemodynamic stress, promoting arrhythmias, myocardial injury and remodeling, MACM, baroreflex desensitization, and a hypercoagulable state, thereby predisposing to LVT and cardioembolic stroke. In parallel, MA-induced gut microbiota dysbiosis and intestinal barrier disruption exacerbate systemic inflammation and alter enteric–vagal signaling, thereby further amplifying neurocardiac dysregulation through the circulation of inflammatory mediators (e.g., TNF-α, LPS). These interacting brain–heart–gut feedback loops collectively establish a maladaptive cycle linking autonomic imbalance, myocardial injury, and systemic inflammation in the pathogenesis of MA-related cardioembolic events. Abbreviations: LPS, lipopolysaccharide; LVT, left ventricular thrombus; MACM, methamphetamine-associated cardiomyopathy; MA, methamphetamine; TMAO, trimethylamine N-oxide; TNF-α, tumor necrosis factor-alpha.

MA abuse disrupts the brain–heart–gut axis through converging mechanisms. Central sympathetic overactivation triggers β-adrenergic signaling and ROS generation in cardiomyocytes; peripheral sympathetic terminals sustain oxidative injury and fibrosis; gut-derived inflammatory mediators amplify myocardial vulnerability; and pathological cardiac afferents destabilize central autonomic control. These mechanisms form an interconnected, multi-level cascade linking central autonomic dysregulation, peripheral neural-myocardial injury, gut-derived inflammation, endothelial dysfunction, and molecular mitochondrial toxicity. Figure 2 summarizes a proposed integrative model of the brain–heart–gut axis in chronic MA exposure leading to ventricular remodeling, thrombogenesis, and cardioembolic stroke.

## 3. Diagnostic Evaluation of MACM and LVT

Patients with suspected MACM commonly present with acute or chronic heart failure, malignant arrhythmias, or a marked reduction in systolic function on initial imaging. Because rapid ventricular dilation and low-flow states are frequent in this population, early evaluation for intracardiac thrombus should be incorporated into the initial diagnostic workup. Accordingly, clinical assessment must address both the severity of cardiomyopathy and the presence of thrombotic complications.

### 3.1. Imaging Modalities

Detection of LVT relies predominantly on cardiac imaging. Transthoracic echocardiography (TTE) remains the first-line modality due to its wide availability, noninvasiveness, and cost-effectiveness; however, its sensitivity is limited, particularly without contrast enhancement. Small, mural, and apex-adherent thrombi may be overlooked, leading to underdiagnosis [29,30].

LVT should be strongly suspected in the presence of severe left ventricular systolic dysfunction, defined as a left ventricular ejection fraction (LVEF) of less than 30–40%, and regional wall motion abnormalities such as apical akinesia or aneurysm. In such cases, advanced imaging techniques with higher spatial resolution are recommended for definitive evaluation [31].

### 3.2. Advanced Imaging Modalities

Cardiac computed tomography (CCT) and cardiac magnetic resonance imaging (CMR) offer superior sensitivity and improved visualization for the detection of LVT. Among these, late gadolinium enhancement CMR (LGE-CMR) is regarded as the diagnostic gold standard, given its high spatial resolution and excellent tissue contrast, which reliably distinguishes thrombus from surrounding myocardium [30].

CCT represents a valuable alternative in clinical settings where echocardiographic findings are inconclusive or when detailed anatomical assessment of the cardiac chambers is required. It also serves as an appropriate option for patients who are unable to undergo CMR due to renal impairment, implanted devices, or other contraindications [32]. In suspected cases of LVT, the use of high-resolution imaging tools for diagnostic confirmation not only improves detection rates but also aids in guiding subsequent anticoagulation strategies.

### 3.3. Laboratory and Biomarker Indicators

Although the definitive diagnosis of LVT relies on imaging, laboratory parameters may provide adjunctive value for risk stratification and longitudinal monitoring. Conventional cardiac biomarkers, including troponin, B-type natriuretic peptide (BNP), and N-terminal pro-B-type natriuretic peptide (NT-proBNP), remain central to assessing the severity of heart failure and disease progression. BNP and NT-proBNP are widely used cardiac biomarkers that reflect myocardial stretch and hemodynamic stress. Inflammatory markers such as high-sensitivity C-reactive protein (hs-CRP) may also reflect ongoing myocardial inflammation or a prothrombotic state [7,33,34,35].

Beyond traditional markers, cancer antigen-125 (CA-125) has emerged as a surrogate indicator of cardiac volume overload and congestion. Elevated CA-125 levels are associated with increased mortality and rehospitalization and correlate with NT-proBNP, LVEF, and pulmonary artery pressure, suggesting value as an indirect marker of pathological hemodynamic stress [34,35].

Despite their clinical utility, no circulating biomarker provides sufficient sensitivity or specificity for definitive LVT diagnosis, underscoring the essential role of imaging confirmation.

### 3.4. Autonomic Nervous Function Assessment

Autonomic evaluation provides a physiologic dimension that complements structural imaging and biomarker analysis in patients with suspected MACM. Heart rate variability (HRV) is the most widely used non-invasive index of autonomic nervous system activity. Reduced HRV has been consistently linked to worsening heart failure, arrhythmogenesis, and increased mortality [36]. In individuals with chronic MA exposure, HRV typically declines with an elevated low-frequency/high-frequency (LF/HF) ratio, indicating sympathetic predominance and parasympathetic withdrawal [19]. Notably, autonomic dysfunction appears to be at least partially reversible. In abstinent MA users, an eight-week aerobic intervention improved the standard deviation of normal-to-normal intervals (SDNN) by approximately 34% and the root mean square of successive differences (RMSSD) by 63%, while reducing the LF/HF ratio, underscoring autonomic plasticity and potential for rehabilitation [37].

BRS offers additional insight into neurocardiac regulation by quantifying cardiac responses to changes in blood pressure. Reduced BRS has been reported in acute ischemic stroke and correlates with worse functional outcomes [38]. Low BRS in combination with abnormal HRV indices may represent a more advanced form of autonomic imbalance and has been associated with a higher risk of adverse cardiovascular events [38,39].

Although the prognostic value of HRV and BRS in MACM-related thromboembolism remains to be fully defined, both metrics demonstrate potential as physiological markers of brain–heart–gut axis dysregulation. Incorporating autonomic parameters into multimodal risk stratification, alongside biochemical and imaging data, may improve early identification of high-risk patients and support individualized management strategies.

## 4. Embolic Patterns and Clinical Presentation: Differences Between MACM and Conventional Cardioembolic Stroke

### 4.1. MACM vs. AF: Clinical and Radiographic Differences

Cardioembolic strokes secondary to MACM exhibit distinct clinical and radiographic features compared to strokes related to AF (Table 1). These events frequently arise during episodes of acute decompensated heart failure, where patients demonstrate marked systolic dysfunction and substantial reductions in LVEF, occasionally progressing to cardiogenic shock [5]. MA users are also predisposed to arrhythmias and infective endocarditis, both of which further heighten ischemic stroke risk. Continued MA use has been associated with increased cardiovascular mortality, recurrent stroke, and rehospitalization, underscoring the link between progressive cardiac dysfunction and embolic events [7,9].

Importantly, MACM-related strokes can occur in the absence of documented arrhythmia. Although MA use increases the risk of tachyarrhythmias, QT prolongation, and AF, prolonged electrocardiography (ECG) monitoring often fails to identify AF episodes. This suggests that LVT in MACM may precipitate stroke without overt electrical abnormalities, reinforcing the need for heightened diagnostic vigilance [9].

The anatomical origin and morphology of thrombi also differ between MACM and AF. AF-related emboli typically arise from the left atrial appendage and are small and friable, leading to multifocal small-vessel occlusions. In contrast, MACM is characterized by pronounced ventricular dilatation and impaired contractility, which promote the formation of larger and structurally stable apical thrombi, and in some cases, biventricular thrombi. These emboli are more likely to cause a single large-vessel occlusion [7,9,14].

Unfavorable prognoses are common in patients with MACM, who often present with chamber enlargement, abnormal cardiac biomarkers, and higher rates of emergency department visits, hospital admissions, and prolonged hospital stays. Cardiac function may improve after MA cessation, whereas continued use is strongly associated with myocardial fibrosis and persistent systolic failure [5,40].

Neuroimaging findings further differentiate these etiologies: AF-related strokes typically appear as scattered multifocal infarcts, whereas MACM-related events more commonly involve a single large cerebral artery with a visible apical thrombus source [7,9,14,41]. These distinctions have significant implications for diagnostic strategy and anticoagulation selection, underscoring the need for personalized management in this population.

### 4.2. Diagnostic Pitfalls: MACM and Embolic Stroke of Undetermined Source (ESUS)

Despite these characteristic clinical and imaging features, MACM-related strokes can be misclassified in clinical practice. In young ischemic stroke patients lacking traditional vascular risk factors, embolic-pattern infarcts may be labeled as ESUS when no embolic source is initially identified. In this context, systematic screening for substance use and toxicology testing is an essential component of evaluating patients for potential MACM-related LVT [9]. Based on established criteria, imaging-confirmed LVT or significant left ventricular systolic dysfunction constitutes a primary cardioembolic source and therefore excludes ESUS categorization [9,41].

**Figure 3:**
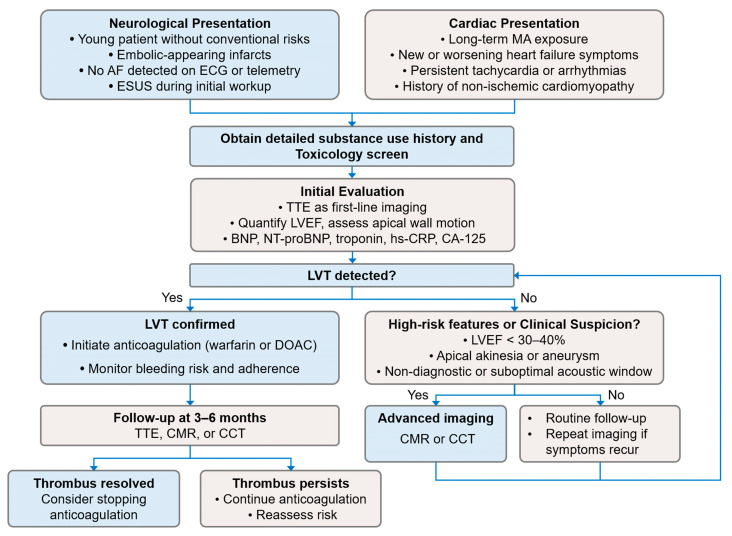
The proposed diagnostic and management algorithm for suspected MACM and LVT. The flowchart illustrates a diagnostic pathway initiated from two distinct clinical scenarios: (**Left**) neurological presentations and (**Right**) cardiac presentations. The algorithm emphasizes the importance of obtaining a detailed substance use history and performing a toxicology screen early in the assessment. Initial cardiac evaluation with TTE is recommended, followed by advanced imaging (CMR or CCT) when clinical suspicion for LVT remains high or when TTE findings are non-diagnostic. Management recommendations, including initiation of anticoagulation and follow-up imaging, are based on established guidelines for LVT detection and treatment. Abbreviations: AF, atrial fibrillation; BNP, B-type natriuretic peptide; CA-125, cancer antigen-125; CCT, cardiac computed tomography; CMR, cardiac magnetic resonance imaging; DOAC, direct oral anticoagulant; ECG, electrocardiogram; ESUS, embolic stroke of undetermined source; hs-CRP, high-sensitivity C-reactive protein; LVEF, left ventricular ejection fraction; LVT, left ventricular thrombus; MA, methamphetamine; NT-proBNP, N-terminal pro-B-type natriuretic peptide; TTE, transthoracic echocardiography.

The diagnostic workflow for suspected MACM differs from the exclusion-based ESUS evaluation and instead benefits proactive, thrombus-directed assessment. At the initial neurological assessment, stimulant use evaluation may be appropriately paired with high-sensitivity cardiac imaging, including CMR or CCT, to detect subtle or mural LVT often missed by routine TTE [5]. This active and confirmatory diagnostic strategy helps reduce the risk of ESUS misclassification, facilitates recognition of LVT arising from severe drug-related cardiomyopathy, and supports timely initiation of anticoagulation therapy (Figure 3).

## 5. Clinical Management of MACM with LVT

Management of MACM with LVT requires simultaneous treatment of heart failure, arrhythmias, and thromboembolism. Clinical care should integrate hemodynamic stabilization, guideline-directed medical therapy, structured substance cessation, and multidisciplinary support.

### 5.1. Cardiac Stabilization: Heart Failure and Arrhythmia Management

Guideline-directed heart failure therapy remains the cornerstone of clinical management. β-adrenergic blockers, angiotensin-converting enzyme inhibitors, angiotensin receptor–neprilysin inhibitors, mineralocorticoid receptor antagonists, and sodium–glucose cotransporter-2 inhibitors should be initiated when hemodynamically tolerated, consistent with guideline-directed therapy for dilated cardiomyopathy and severe systolic dysfunction [5,42]. Diuretics provide symptomatic relief from congestion, while short-term inotropes or vasopressors may be required in cases of cardiogenic shock. Benzodiazepines are favored for managing sympathetic overactivity associated with stimulant withdrawal or severe agitation [5,42].

Arrhythmia management is critical, as ventricular tachyarrhythmias and AF are frequently encountered in MACM [9]. β-blockers serve dual roles in rate control and sympathetic suppression, whereas refractory cases may require amiodarone or catheter ablation [5].

Intra-aortic balloon pump counterpulsation, Impella microaxial flow pumps, and veno-arterial extracorporeal membrane oxygenation can serve as a bridge to myocardial recovery in acute cardiogenic shock [5,7]. The presence of LVT warrants prompt anticoagulation to mitigate embolic complications.

### 5.2. Anticoagulation Strategies for MACM with LVT

Evidence specific to anticoagulation in MACM complicated by LVT remains scarce, with current management generally extrapolated from studies of LVT in other forms of non-ischemic cardiomyopathy and post-myocardial infarction populations [5,7]. In non-ischemic cardiomyopathy, routine oral anticoagulation for primary prevention is not recommended for patients in sinus rhythm; anticoagulation is generally reserved for confirmed LVT or selected high-risk subtypes such as left ventricular noncompaction [12]. Accordingly, in MACM, reduced LVEF alone without evidence of thrombus is not an indication for routine anticoagulation. Individualized consideration is reasonable when marked regional wall motion abnormalities or apical aneurysms raise concern for stasis [5,7,12].

Vitamin K antagonists, particularly warfarin, have historically served as standard therapy for LVT. Recent systematic reviews and meta-analyses indicate that direct oral anticoagulants (DOACs) demonstrate comparable efficacy to warfarin in thrombus resolution, prevention of systemic embolism, and overall safety, with a potentially lower bleeding risk in selected populations [12,43,44].

LVT morphology may add complexity to treatment decisions; however, current evidence does not support morphology-specific anticoagulation strategies. Newly identified mural and protuberant thrombi are typically managed similarly with oral anticoagulation, and no robust data exist to guide differences in drug selection [12].

Substance use, irregular follow-up, and inconsistent adherence complicate anticoagulant selection in MACM. DOACs are often favored when international normalized ratio (INR) monitoring is unfeasible or when frequent laboratory testing is uncertain [12,45].

Overall, DOACs represent a reasonable alternative to warfarin, particularly when INR control is suboptimal. However, no specific clinical or imaging-based thresholds exist to favor DOACs in MACM-related LVT. Treatment decisions should be individualized, taking into account thrombus characteristics, bleeding risk, likelihood of adherence, and feasibility of imaging follow-up, ideally within the context of shared decision-making.

### 5.3. Anticoagulation Duration and Monitoring

Duration and monitoring strategies for MACM-related LVT have not been prospectively studied. The 2022 AHA scientific statement recommends anticoagulation for 3 to 6 months with serial imaging to confirm thrombus resolution. Discontinuation may be considered if the thrombus has completely resolved and high-risk features, such as severely reduced LVEF or persistent regional wall motion abnormalities, are absent. Conversely, prolonged therapy is warranted if thrombus persists or high-risk structural abnormalities remain [5,12] (Figure 3).

When embolic events occur despite therapeutic anticoagulation, rigorous reassessment is required. Verifying anticoagulant compliance and screening for substance relapse are the requisite initial steps, as nonadherence is the predominant cause of therapeutic failure in this population [5,7]. If compliance is confirmed and events persist, repeat cardiac imaging is warranted to reassess thrombus burden, mobility, and structural status. Treatment modifications with anticoagulant class-switching should follow standard secondary stroke prevention guidelines: transitioning from DOACs to warfarin, or from warfarin (despite therapeutic INR) to low-molecular-weight heparin or an alternative DOAC [5,12,46]. Use of concomitant antiplatelet is discouraged unless clearly indicated (e.g., recent coronary stent placement) to avoid excess bleeding risk [46].

For acute ischemic stroke, management follows standard guidelines, prioritizing revascularization (intravenous thrombolysis or mechanical thrombectomy) when eligibility criteria are met, with subsequent cautious blood pressure control to reduce reperfusion injury [46].

In the long term, patients with persistent LVEF ≤ 35% despite optimized medical therapy and MA abstinence, implantable cardioverter–defibrillator evaluation is warranted to mitigate the risk of sudden cardiac death [5].

Recurrent embolism in MACM often reflects persistent dysfunction, thrombotic burden, and unstable adherence. Management must integrate anticoagulant strategy, structural heart care, and substance use intervention through coordinated, multidisciplinary care [5,7,9].

### 5.4. Unique Challenges and Integrated Care Considerations

MACM-related stroke predominantly affects young adult males, who frequently demonstrate ongoing MA use, polysubstance exposure, unstable housing, unemployment, and poor medical adherence [5,7]. These factors, together with a higher prevalence of hypertension and alcohol misuse, contribute to attenuated myocardial recovery, recurrent decompensation, and suboptimal neurorehabilitation outcomes [42,47].

Absolute MA abstinence is a critical component of therapy, as sustained cessation has been associated with partial or even full recovery of cardiac function. Myocardial function can improve significantly after months of sobriety, especially if instituted before irreversible fibrosis has developed [7,48,49,50]. Adherence to anticoagulation is undermined by socioeconomic and behavioral instability. Furthermore, comorbidities elevate the risk of major bleeding events such as gastrointestinal or intracranial hemorrhage [9,51,52]. In this context, pharmacologic management alone is insufficient to achieve durable clinical benefit.

Multidisciplinary care is imperative. Neuromodulatory interventions, particularly repetitive transcranial magnetic stimulation (rTMS) and intermittent theta burst stimulation (iTBS) targeting the dorsolateral prefrontal cortex, have demonstrated reductions in craving intensity and improvements in executive control in randomized clinical trials [53,54]. Complementary psychological therapies, including cognitive behavioral therapy, contingency management, mindfulness-based interventions, and group counseling, have been shown to improve adherence and support sustained abstinence. Social work involvement is similarly critical for facilitating access to housing, employment resources, and community support networks, thereby enhancing stability and continuity of care [55,56].

The long-term prognosis in MACM is closely tied to psychosocial factors. Integrating cardiovascular and cerebrovascular care with behavioral and social interventions offers the best opportunity to reduce recurrence and improve outcomes.

## 6. Clinical Case Illustration

A 34-year-old man with no significant past medical history presented to the emergency department with sudden-onset left-sided weakness and dysarthria. Upon admission, his blood pressure was 153/99 mmHg, and his heart rate was 101 beats per minute. Neurological examination revealed right gaze deviation, left visual field deficit, left facial droop, and severe left hemiplegia. The initial National Institutes of Health Stroke Scale (NIHSS) score was 16, consistent with severe ischemic stroke.

**Figure 4:**
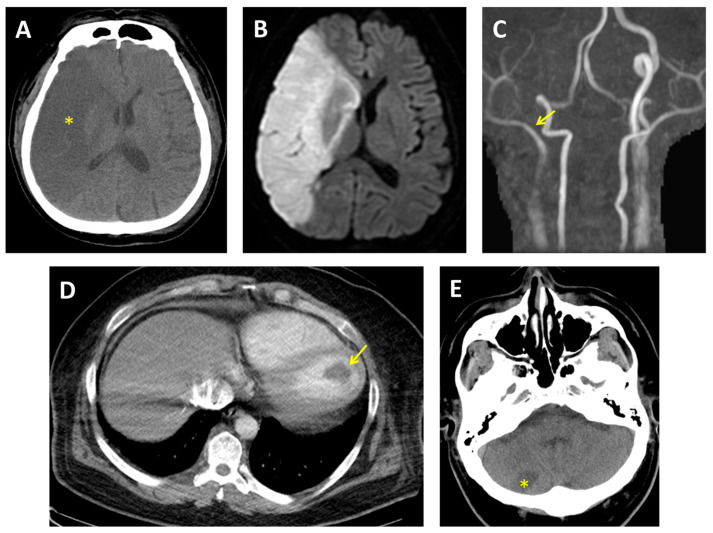
(**A**) Brain CT initially revealed a right middle cerebral artery territory with a large hypodensity (star). (**B**) Brain MRI showed hyperintensity on DWI in the same area. (**C**) MRA showed total occlusion of the right internal carotid artery (arrow). (**D**) Contrast-enhanced CT of the chest revealed a left ventricular thrombus (arrow). (**E**) Follow-up brain CT showed a new hypodensity in the right cerebellum (star). CT, computed tomography; DWI, diffusion-weighted imaging; MRA, magnetic resonance angiography; MRI, magnetic resonance imaging.

Non-contrast brain CT revealed a large hypodense lesion in the right middle cerebral artery (MCA) territory (Figure 4A). Subsequent diffusion-weighted magnetic resonance imaging (DWI) confirmed acute ischemic infarction (Figure 4B), and magnetic resonance angiography (MRA) demonstrated complete occlusion of the right internal carotid artery (ICA) (Figure 4C). Urine toxicology was positive for MA, and the patient reported daily use for over five years.

Cardiothoracic CT revealed a large thrombus within the left ventricular cavity (Figure 4D). TTE showed four-chamber enlargement and severe systolic dysfunction, with a LVEF of 24%, consistent with MACM. Continuous 24 h ECG monitoring did not detect AF. Laboratory tests revealed an elevated Factor VIII level at 257% (reference range: 60–150%) and a CA-125 level of 168.8 U/mL (normal range: <35.0 U/mL), indicating a hypercoagulable state and increased cardiac filling pressures.

On hospital day 5, the patient developed a new right cerebellar infarction (Figure 4E), indicating ongoing embolic activity. Initial anticoagulation with warfarin was complicated by gastrointestinal bleeding, prompting a switch to rivaroxaban, which was continued without further thromboembolic or bleeding events during hospitalization.

He was discharged on day 26 with persistent neurological deficits (NIHSS 18, modified Rankin Scale [mRS] of 4), requiring 24 h care, and was transferred to a long-term care facility. At the three-month follow-up, his mRS score remained unchanged, and no recurrent stroke events were reported. Given severe post-stroke disability and transition to long-term care, no follow-up cardiac imaging was obtained.

This case highlights the substantial cardiovascular and cerebrovascular risk associated with MACM. In young patients with ischemic stroke, the coexistence of severe left ventricular systolic dysfunction and a hypercoagulable profile should prompt consideration of drug-related cardiomyopathy as a cardioembolic source. Cardiac and thoracic imaging facilitates early identification of LVT, particularly when arrhythmia is absent. The markedly elevated CA-125 level in this patient may reflect cardiac congestion and warrants further investigation as a potential adjunctive biomarker for disease severity and monitoring.

The successful transition from warfarin to a DOAC in the setting of gastrointestinal bleeding illustrates a feasible alternative in high-risk patients, despite the lack of randomized trial data in MACM-related LVT. This case underscores the importance of individualized anticoagulation strategies and highlights the urgent need for dedicated clinical evidence to guide optimal management and long-term prognosis in this growing population.

## 7. Literature Context: Published Evidence on MACM with Thrombotic Complications

Current evidence on MACM remains limited and heterogeneous, with most publications consisting of small observational cohorts supplemented by single-patient case reports. Using a predefined search strategy and selection criteria (Supplementary Table S1), we synthesized these data into Table 2, which integrates both cohort-level findings and detailed case reports. [7,10,14,45,48,49,50,57,58,59,60,61,62,63,64,65,66,67,68,69].

Across published studies, affected individuals are typically young, predominantly male, and frequently demonstrate marked left ventricular systolic dysfunction, with reported mean LVEF ranging from 19% to 30%. Studies incorporating systematic cardiac imaging have identified a meaningful prevalence of LVT, approximately one-third of patients in Schürer (2017) [7] and 12% in Zhao (2020) [49], raising concern that the true burden may be underestimated in settings where surveillance imaging is not routinely performed. Abstinence consistently correlates with improved outcomes, including higher follow-up LVEF, fewer heart failure hospitalizations, and lower mortality [48,49,50,58]. Even among individuals with severe systolic dysfunction, abstinence was associated with meaningful LVEF recovery [50]. In stroke populations, as demonstrated by Lee et al. (2024) [10], MACM was strongly associated with impaired systolic function, LVT burden, and cardioembolic mechanisms, reinforcing the heart–brain interaction in this disease process.

The case-level data in Table 2 further expand this spectrum, illustrating infrequent but clinically important presentations, including biventricular thrombi [60,65], massive apical thrombi [65], and recurrent stroke occurring in the context of MACM [14,68]. These reports also highlight the broader systemic embolic burden associated with MACM, encompassing aortic and limb ischemia [61,66,69], renal infarction [61,69], pulmonary embolism [60,65], and cardiogenic shock resulting from extensive ventricular thrombi [61]. Management strategies varied substantially, ranging from unfractionated heparin, DOACs, and warfarin bridging to mechanical thrombectomy and circulatory support, reflecting the absence of consensus on antithrombotic selection, timing, or duration. Follow-up imaging protocols were inconsistently reported, limiting evaluation of thrombus resolution, embolic recurrence, and criteria for anticoagulation tapering.

## 8. Limitations

This review is narrative in scope and therefore limited to observational-level evidence. Interpretation of existing studies is constrained by substantial methodological heterogeneity. Diagnostic criteria for MACM are not standardized, and the use of advanced cardiac imaging varies widely across reports. Anticoagulation practices also demonstrate considerable variability in drug selection, treatment duration, and criteria for discontinuation, resulting in inconsistent outcome reporting.

Long-term follow-up data are scarce, particularly regarding thrombus resolution, recurrence, bleeding risk, and outcomes after withdrawal of therapy. Definitions of embolic endpoints and clinical deterioration differ between studies, further preventing pooled analysis or quantitative synthesis.

Given these limitations, current knowledge relies heavily on small cohorts and case-level evidence. The available literature is also likely affected by publication bias, as severe or recurrent embolic events are more frequently reported, whereas patients with milder ventricular dysfunction or transient thrombi may be underrepresented. Prospective registries, standardized imaging protocols, and harmonized outcome definitions will be crucial for generating MACM-specific evidence and informing future clinical practice.

## 9. Conclusions

MACM has emerged as an important cause of heart failure and cardioembolic stroke in younger adults, yet it remains underrecognized in clinical practice. Chronic MA exposure disrupts regulatory pathways within the brain–heart–gut axis, leading to dysregulation of dopaminergic and noradrenergic signaling. This results in autonomic imbalance and sustained sympathetic overactivation. Additionally, alterations in gut microbiota and systemic inflammation promote oxidative stress, microvascular injury, and a prothrombotic milieu. These neuro-immune mechanisms contribute to myocardial fibrosis, ventricular remodeling, and impaired intracardiac flow, thereby increasing the propensity for LVT formation and systemic embolization.

From a diagnostic perspective, MACM should be considered in the differential diagnosis of cardioembolic or cryptogenic stroke in young adults, particularly in the presence of positive toxicology screens or unexplained left ventricular systolic dysfunction. Early utilization of high-resolution cardiac imaging may improve the detection of LVT and guide anticoagulation decisions. Although DOACs have demonstrated favorable safety and efficacy profiles in mixed-etiology LVT, no randomized evidence exists specifically for MACM, and treatment decisions currently require individualized risk assessment and close imaging surveillance.

Clinical outcomes in MACM are also heavily influenced by behavioral and psychosocial factors, including continued substance use, limited healthcare access, inadequate addiction support, and poor medication adherence. These factors contribute to persistent ventricular dysfunction, interruption of anticoagulation, and recurrent thromboembolism, underscoring that cardiac therapy alone is insufficient. Integrated care models centered on addiction treatment, longitudinal follow-up, and supportive services are essential to achieve durable improvement.

Early recognition, cessation of MA use, individualized anticoagulation guided by multimodal cardiac imaging, and integration of addiction treatment into stroke and heart failure pathways are essential to improving outcomes. Prospective registries and multicenter studies are needed to establish MACM-specific risk stratification tools, standardized diagnostic protocols, and comprehensive treatment strategies.

## Figures and Tables

**Table 1:** Key distinctions between MACM- and AF-related cardioembolic stroke.

Feature	MACM-Related Stroke	AF-Related Stroke
Underlying mechanism	MA-induced toxic cardiomyopathy with severe LV dysfunction	Atrial arrhythmia causing stasis in the LA
Typical cardiac finding	LV dilatation, apical thrombus, may occur without AF	AF on ECG; LA appendage thrombus
Thrombus location	LV (often apical)	Left atrial appendage
Infarct pattern	Single large-vessel occlusion	Multiple small or scattered infarcts
Clinical context	Occurs during decompensated heart failure or MACM	Occurs during or after an AF episode
Prognosis	Worse outcomes; improvement with MA cessation	Variable; depends on AF and anticoagulation control

Abbreviations: AF, atrial fibrillation; ECG, electrocardiography; LA, left atrium; LV, left ventricle; MA, methamphetamine; MACM, methamphetamine-associated cardiomyopathy.

**Table 2:** Published cohorts and case-level reports of MACM, with associated cardiac or systemic thrombotic complications and clinical outcomes.

Author (Year)	Population/Demographics	LVEF	LVT	Stroke/Embolic Event	Treatments	Clinical Outcome
Yew (2014) [59]	33-year-old male	Severely reduced	No (spontaneous echo contrast)	Left anterior circulation infarction	Antiplatelets; statin; antihypertensives	Abstinent; neurologic recovery; NYHA I at 6 months; LVEF normalized at 1 year.
Janardhanan (2016) [60]	35-year-old male	23%	Yes—LV and RV	Pulmonary embolism	IV heparin; warfarin; ticagrelor; HF therapy	Abstinent; LVEF improved to 30% at 3 months; no recurrent embolic events.
Sliman (2016) [48]	141 MA-associated HF (mean 52 ± 9 years; 79% male)	Mean 29.9%	Not reported	Not reported	Standard HF therapy	Abstinence associated with improved NYHA class, better functional status, and trend toward LVEF recovery.
Voskoboinik (2016) [57]	20 MACM (mean 35 ± 9 years; 70% male)	Mean 19.7%	Not reported	Not reported	Inotropes; ICU care; ECMO/IABP; GDMT	Six of 19 achieved LVEF normalization within 6 weeks; reverse-Takotsubo morphology associated with early recovery.
Schürer (2017) [7]	30 MACM (mean 30 ± 9 years; 77% male)	Mean 19%	Yes—single or multiple (10 cases)	Yes—non-fatal stroke reported (composite outcome)	ECMO/IABP; valve surgery; ICD; wearable cardioverter–defibrillator; anticoagulation; GDMT	Abstinence associated with higher follow-up LVEF and fewer composite adverse events (death, nonfatal stroke, and rehospitalization).
Eliveha (2019) [61]	24-year-old female	15%	Yes—single	Aortic occlusion; superior mesenteric artery thrombus; bilateral renal infarcts; limb ischemia	Surgical thrombectomy; vasopressors; supportive care	Not abstinent; died from cardiogenic shock with end-organ failure.
Navid (2019) [62]	34-year-old male	10–15%	No (spontaneous echo contrast)	No embolic event; spontaneous coronary artery dissection	GDMT; aspirin; clopidogrel	Not abstinent; persistent severe LV dysfunction; ineligible for transplant.
Schwarzbach (2020) [45]	23-year-old male	20%	No	Not reported	Inotropes; GDMT; wearable cardioverter–defibrillator vest	Not abstinent; no LV recovery; HF readmission.
Zhao (2020) [49]	357 MACM (mean 47 ± 10 years; 83% male)	≤40%	12% overall	Not reported	GDMT; ICD/CRT when indicated	Abstinence was associated higher LVEF and lower HF hospitalization and mortality.
Bhatia (2021) [50]	56 MACM: reduced LVEF group (n = 28, 51 ± 9 years, 82% male); preserved LVEF group (n = 28, 50 ± 8 years, 61% male)	Mean 30.2% in reduced LVEF group; mean 66.2% in preserved LVEF group	Not reported	Not reported	GDMT	Abstinence associated with improved LVEF and fewer HF hospitalizations.
Patel (2021) [63]	30-year-old female	20%	Yes—single	Stroke-like symptoms without infarction	Apixaban; GDMT	Neurologic symptoms improved; discharged on apixaban; lost to follow-up.
Bhatia (2022) [58]	31 MA-associated preserved LVEF (mean 49 ± 10 years; 55% male)	Median 66%	Not reported	Not reported	Standard HF therapy	Abstinence at 1 year associated with improved GLS and reduced HF admissions.
Del Rio-Pertuz (2022) [64]	33-year-old female	3–20%	Yes—multiple	STEMI due to coronary embolism	Anticoagulation; GDMT; mechanical aspiration	Persistent low LVEF and LVT.
Zaidi (2022) [65]	48-year-old female	18%	Yes—multiple; LV and RV	Pulmonary embolism	IV heparin; warfarin	Not abstinent; HF readmission; decreased LVT burden; RV thrombus resolution or embolization.
Dhaliwal (2023) [14]	60-year-old male	20–25%	Yes—LV and RV	Cortical infarcts	IV thrombolysis; heparin; GDMT; rivaroxaban	Abstinent; NIHSS 0 at discharge; medication compliant.
Lee (2024) [10]	46 MA-associated acute ischemic stroke (mean 52.8 ± 9.6 years; 78.3% male); 14 with MACM	Mean 26% in MACM	Yes—LV thrombus (4 patients)	All patients had acute ischemic stroke	GDMT; antithrombotic therapy based on stroke guidelines; revascularization when indicated	mRS 0–2 at 3 months in 64.3% of MACM subgroup; 2 deaths; MACM strongly associated with cardioembolic stroke (92.9%).
Newman (2024) [66]	39-year-old male	14% initially; 8% after deterioration	Yes—LV and RV	Aortic occlusion; bilateral limb ischemia	Aortoiliac thrombectomy; IV heparin; rivaroxaban; GDMT	Not abstinent; successful reperfusion; neurologic recovery; ambulatory.
Zuern (2024) [67]	49-year-old male	35%	Yes—single	No embolic event	Oral anticoagulation; GDMT	LVT resolved; LVEF normalized.
Fujioka (2025) [68]	54-year-old male	24.1%	Yes—single	Left internal carotid artery occlusion	Warfarin; UFH bridging; mechanical thrombectomy; diuretics; antihypertensives; antiseizure medication	Complete reperfusion; cerebral hyperperfusion; mRS 3 at day 90; LVEF improved to 53%.
Moayerifar (2025) [69]	Early-40 s male	5–10% initially; 10–15% at discharge	Yes—giant thrombus	Left common iliac artery thrombosis; renal infarct; transient dysarthria	IV heparin; warfarin; GDMT; iliac artery stenting	Abstinent; LVT markedly reduced with improved LV size at 3 months; limb ischemia improved; LVEF 35%.

Values are extracted from published observational cohorts reporting clinical presentation, management strategies, and outcomes in patients with MACM. Abbreviations: CRT, cardiac resynchronization therapy; ECMO, extracorporeal membrane oxygenation; GDMT, guideline-directed medical therapy; GLS, global longitudinal strain; HF, heart failure; IABP, intra-aortic balloon pump; ICD, implantable cardioverter–defibrillator; ICU, intensive care unit; IV, intravenous; LV, left ventricle; LVEF, left ventricular ejection fraction; LVT, left ventricular thrombus; MACM, methamphetamine-associated cardiomyopathy; mRS, modified Rankin Scale; NIHSS, National Institute of Health Stroke Scale; NYHA, New York Heart Association; RV, right ventricle; STEMI, ST Elevation Myocardial Infarction; UFH, unfractionated heparin.

## Data Availability

All data cited in this review were obtained from publicly available, peer-reviewed sources indexed in PubMed and Scopus. No new data were generated or analyzed in the course of this work. Therefore, data sharing is not applicable.

## Supplementary Materials

The following supporting information can be downloaded at https://www.mdpi.com/article/10.3390/ijms262411908/s1.

## Abbreviations

The following abbreviations are used in this manuscript:

AF	Atrial fibrillation
BNP	B-type natriuretic peptide
BRS	Baroreflex Sensitivity
CA-125	Cancer antigen-125
CCT	Cardiac computed tomography
CMR	Cardiac magnetic resonance imaging
CNS	Central nervous system
DOAC	Direct oral anticoagulant
DWI	Diffusion-weighted imaging
ENS	Enteric nervous system
ESUS	Embolic Stroke of Undetermined Source
GDMT	Guideline-directed medical therapy
HRV	Heart rate variability
LPS	Lipopolysaccharide
LVEF	Left ventricular ejection fraction
LVT	Left ventricular thrombus
MA	Methamphetamine
MACM	Methamphetamine-associated cardiomyopathy
MRA	Magnetic resonance angiography
MRI	magnetic resonance imaging
mRS	Modified Rankin Scale
NIHSS	National Institutes of Health Stroke Scale
NT-proBNP	N-terminal pro-B-type natriuretic peptide
ROS	Reactive oxygen species
TMAO	Trimethylamine N-oxide
TTE	Transthoracic echocardiography
