# Supplementary material for: Methamphetamine-Associated Cardiomyopathy and Cardioembolic Stroke: Brain–Heart–Gut Axis Crosstalk, Diagnostic Strategies, and Anticoagulation Challenges
Source: Int J Mol Sci. 2025 Dec 10;26(24):11908. doi: 10.3390/ijms262411908 (PMC12732741; doi:10.3390/ijms262411908)
Supplement: Supplementary file 1 [file ijms-26-11908-s001.zip › ijms-4015557-supplementary.pdf]

Supplementary Table S1. Literature search strategy and selection criteria

Component	Description
**Search period**	January 1, 1990 – November 8, 2025
**Language restriction**	English only
**Search type**	Structured literature review
**Search keywords and Boolean strategy**	**Pubmed**<br>(("methamphetamine"[tiab] OR "crystal meth"[tiab])<br>AND ("cardiomyopathy"[tiab] OR "heart failure"[tiab] OR MACM[tiab] OR "dilated cardiomyopathy"[tiab])<br>AND ("stroke"[tiab] OR "thrombus"[tiab] OR "embolism"[tiab] OR thrombosis[tiab] OR "left ventricular thrombus"[tiab] OR LVT[tiab]))<br><br>**Scopus**<br>(TITLE-ABS-KEY(methamphetamine OR "crystal meth")<br>AND TITLE-ABS-KEY("cardiomyopathy" OR "heart failure" OR MACM OR "dilated cardiomyopathy")<br>AND TITLE-ABS-KEY(stroke OR thrombus OR embolism OR thrombosis OR "left ventricular thrombus" OR LVT)<br>AND TITLE-ABS-KEY(human OR humans OR patient OR case)<br>AND NOT TITLE-ABS-KEY(mouse OR mice OR rat OR murine OR rabbit OR canine OR animal*))
**Screening process**	1. Titles and abstracts screened for relevance<br>2. Full-text review for eligibility<br>3. Final inclusion by consensus<br>4. Reference lists of eligible articles were additionally screened to identify missed publications.
**Inclusion criteria**	1. Published between 1990 and 2025<br>2. English language<br>3. Human subjects<br>4. Case reports, case series, or observational cohorts<br>5. Definitive diagnosis of MACM confirmed by echocardiography, cardiac CT, or cardiac MRI<br>6. Reported patient-level clinical or imaging data on left ventricular

		dysfunction and/or thromboembolic complications
**Exclusion criteria**	1.	Non-English publications
	2.	Experimental/animal studies
	3.	Reviews without primary patient data
	4.	Cases lacking cardiac imaging confirmation of MACM
	5.	Cases without clinical information on left ventricular function or thrombotic events
**Primary extracted variables**	Demographics, LVEF, presence of LVT or systemic embolism, treatments, clinical outcomes	
**Output tables**	**Table 2.** Published cohorts and case-level reports of MACM, with associated cardiac or systemic thrombotic complications and clinical outcomes	

Search updated through November 8, 2025. Reference lists of eligible articles were additionally screened to identify missed publications.

Abbreviations: CT, computed tomography; MRI, magnetic resonance imaging; LVEF, left ventricular ejection fraction; LVT, left ventricular thrombus; MACM, methamphetamine-associated cardiomyopathy.
